# The Patterns of Graded Psychological Nursing Care for Patients after Cardiothoracic Surgeries

**Published:** 2017-07

**Authors:** Hui SUN, Hongyu LIU, Jingbo LI, Xiaochun WANG

**Affiliations:** 1. Operating Room, The First Affiliated Hospital of Harbin Medical University, Harbin, Heilongjiang Province, China; 2. Dept. of Cardiac Surgery, The First Affiliated Hospital of Harbin Medical University, Harbin, Heilongjiang Province, China; 3. Dept. of Nursing, The First Affiliated Hospital of Harbin Medical University, Harbin, Heilongjiang Province, China

**Keywords:** Graded psychological nursing care, SCL-90 score, Pittsburgh sleep quality index

## Abstract

**Background:**

To investigate the interventional efficacy and clinical significance of graded psychological nursing care for patients who have undergone cardiothoracic surgery by providing graded psychological nursing care for these patients according to the results of their psychological evaluation.

**Methods:**

In this interventional study, 110 patients who had undergone cardiothoracic surgery between 2014 and 2015 in the First Affiliated Hospital of Harbin Medical University, Harbin, Heilongjiang Province, China, were enrolled. We divided them into two groups of 55 patients each, namely, a control group and a treatment group. For patients in the control group, we applied regular psychological nursing care; those in the treatment group were further divided into three different psychological grades after being assessed using Symptom Checklist 90 (SCL-90) and the Pittsburgh Sleep Quality Index (PSQI); patients at each grade were treated by graded psychological nursing care in addition to regular psychological nursing care.

**Results:**

Significant decreases, with statistically significant differences (*P*<0.01), were observed in the SCL-90 and PSQI scores of patients in the treatment group as compared with the control group. Patients in the treatment group, who underwent graded psychological nursing care according to their varying psychological conditions, showed better improvement in their post-surgery emotional state and sleep quality than those in the control group, thus indicating the great significance of graded psychological nursing care in clinical practices.

**Conclusion:**

Applying graded psychological nursing care in post-operation cardiothoracic patients improved nursing care efficiency and alleviated patients’ negative feelings. Therefore, this type of nursing care should be further promoted and utilized in clinical practice for effective rehabilitation of patients.

## Introduction

With continuous developments in medicine, treatment has focused not only on patients’ physical recovery but also on the achievement of a healthy psychological state. Psychological nursing care refers to medical professionals providing positive psychological interventions for patients after surgery to help them acquire and maintain a good psychological state to facilitate rehabilitation (1).

Surgery is the most frequently used method in clinical practice in general. However, due to the high risk associated with it, it can cause patients to suffer from negative emotions such as fear, anxiety, or even depression. In fact, the greater the difficulty and risk of the thoracic surgery, the more patients are susceptible to negative emotions that affect their post-operation rehabilitation and the treatment therapeutic effect (2). Thus, individual psychological nursing care is of great significance for these patients. Although ordinary psychological nursing care has been designed for the majority of patients, it shows a lack of specificity. In contrast, as graded nursing care is formulated according to the patient’s current psychological condition (3), it could produce more significant efficacy, particularly for patients undergoing cardiothoracic surgery.

In this study, we selected 110 cardiothoracic patients between 2014 and 2015 to compare the efficacies of conventional and graded psychological nursing care.

## Materials and Methods

In this interventional study, we randomly selected 110 cardiothoracic patients between 2014 and 2015 from the First Affiliated Hospital of Harbin Medical University, Harbin, Heilongjiang Province, China.

All selected patients voluntarily participated in this study, which was approved by the Ethics Committee of the First Affiliated Hospital of Harbin Medical University.

We excluded patients who were suffering from mental diseases before admission or those unwilling to cooperate with the therapies (4). Using a random number table, all patients were divided into two groups of 55 patients each: a control group and a treatment group. Among all patients, 68 males and 42 females participated, with an average age of 32.7 ± 4.5 years. The control group included 32 males and 23 females, with an average age of 31.9 ± 6.1 years; the treatment group included 36 males and 19 females, with an average age of 31.1 ± 7.1 years. No statistically significant differences were observed on comparing patients’ genders and ages between the two groups (*P* > 0.05).

### Methods

Patients in the treatment group were assessed according to the Symptom Checklist 90 (SCL-90) and Pittsburgh Sleep Quality Index (PSQI) (5). The former included the following ten factors: senses, feelings, thought, consciousness, behavior, lifestyle, personal relationship, diet, and sleep. A negative correlation was observed between the SCL-90 score and the psychological state. PSQI was utilized for assessing and scoring patients’ sleep duration, sleep quality, getting out of bed at night and daytime mental state for nearly 30 days. The total score of PSQI was set as 21 points; patients with lower scores enjoyed better sleep quality. The patients and medical professionals accomplished the assessment together.

The participating cardiothoracic surgery patients were divided into three grades according to the assessment results. The nursing care standards for patients of each grade are shown in Table 1.

**Table 1:** Nursing care standards for patients in three grades

**Items**	**Third-class nursing care standard**	**Second-class nursing care standard**	**First-class nursing care standard**
SCL-90	<2 points	2–3.5	>3.5 points
Scores of factor	The score of each factor<2 points	Less than 7 factors with a score of more than 2 points; no factor with a score of more than 3 points	More than 7 factors with a score of more than 2 points; single factor with a score of more than 3 points
PSQI	<7 points	7–15 points	>15 points
Sleep quality	Good	Poor	Very poor or no sleep
Other	Patients were positive and optimistic for rehabilitation	Patients had some negative feelings, such as being slightly anxious or depressed	Patients had obvious abnormal feelings

In the treatment group, third-, second-, and first-class nursing care was performed for 30, 19, and 6 patients, respectively. Third-class psychological nurse management: Medical professionals established good relationships with patients, tried their best to perceive their negative feelings by talking to them, provided them with various kinds of psychological support and communication in combination with conventional psychological nursing care, and encouraged them to be positive and confident of their rehabilitation (6).

Second-class psychological nurse management: Medical professionals provided individual psychological guidance based on third-class psychological nurse management owing to patients’ individual differences and varying post-operative negative feelings. Medical professionals spent time in communicating with patients daily, and, if necessary, arranged various kinds of entertainment, which included telling stories, listening to music, or watching TV, to alleviate patients’ abnormal feelings of anxiety or depression. Further, medical professionals also communicated with the patients’ families to gather an understanding of the patients’ psychological condition indirectly and informed them of regular psychological counseling methods.

First-class psychological nurse management: On the basis of second-class psychological nurse management, medical professionals qualified for psychological counseling as they had a deeper understanding of patients’ psychological knowledge, closely observed their psychological condition, communicated with them at a definite time every day, helped them to comprehend the rehabilitation process, encouraged them to positively cooperate with treatment without any abnormal feelings, and provided psychological support to them. When patients became overwhelmed, medical professionals immediately intervened through counseling, communicating, or, if necessary, inviting psychological experts to attend group consultation. Medical professionals had to classify any negative feelings perceived in patients, identify the origin of such feelings, and enact corresponding procedures. For example, if patients were experiencing pain due to the surgery, the medical professionals were to have pain relievers administrated, comfort and enlighten patients, and guide them to cooperate with the therapies positively. Patients were provided with psychological counseling and nursing care to varying degrees based on their demands, and medical professionals who provided the former support were required to improve their comprehensive ability of psychological nursing care. Thus, the first-class procedures focused on stipulating specific solutions for patients’ abnormal feelings, which were more individual than regular psychological nursing care. At the time of discharge, patients in both groups were assessed using SCL-90 and PSQI again and the results of assessments after surgery and before discharge were compared.

Patients in both groups assessed the comfort level of clinical nursing care. The total score was set as 10 points and the assessment was conducted in the form of a questionnaire containing ten questions, with 1 or 0 points being given for each question. The ten questions were as follows: 1. Did you experience any alleviation in symptoms such as chest distress or shortness of breath? 2. Did you experience any discomfort due to the regular diet? 3. Are you satisfied with the patterns of your nursing care? 4. Are you satisfied with the ward environment? 5. Are you satisfied with your sleep quality? 6. Are you satisfied with the work of the nurse responsible for you? 7. Are you willing to go to this hospital for treatment in the future? 8. Has there been any improvement in your diseases? 9. Are you confident about your recovery? 10. Are you willing to continue to accept nursing intervention?

### Statistical methods

The data was statistically analyzed using SPSS 21.0 (Chicago, IL, USA). The chi-squared test and *t* test were applied to compare counted and measured data between the two groups, respectively. Differences with *P* < 0.05 were considered statistically significant.

## Results

### Comparison of SCL-90 and PSQI scores between the two groups

For the patients in the control group, the average SCL-90 and PSQI scores before surgery were, respectively, 3.11 ± 0.51 and 3.66 ± 0.47; for the patients in the treatment group, these two scores were, respectively, 2.98 ± 0.51 and 3.75 ± 0.49. Statistically significant differences were observed in both comparisons of SCL-90 and PSQI scores between the two groups (*P* < 0.05) (Table 2).

**Table 2:** Comparison of the SCL-90 and PSQI scores of the enrolled patients in both groups

**Groups**	**Cases**	**SCL-90 scores**	**PSQI**
Control group	55	3.11 ± 0.51	3.66 ± 0.47
Treatment group	55	2.98 ± 0.51	3.75 ± 0.49
*P* value	<0.05	<0.05	<0.05

### Comparison of patients-surgery SCL-90 and PSQI scores between groups psychological counselin (x̄± s)

For the patients in the control group, the average SCL-90 and PSQI scores before discharge were, respectively, 2.98 ± 0.43 and 3.27 ± 0.49; for the patients in the treatment group, the average SCL-90 and PSQI scores before discharge were, respectively, 1.77 ± 0.45 and 2.88 ± 0.46. Statistically significant differences were observed in both comparisons of post-surgery SCL-90 and PSQI scores between the two groups (*P* < 0.05) (Table 3).

**Table 3:** Comparison of the SCL-90 and PSQI scores of patients before discharge in both groups

**Groups**	**Cases**	**SCL-90 scores**	**PSQI**
Control group	55	2.98 ± 0.43	3.27 ± 0.49
Treatment group	55	1.77 ± 0.45	2.88 ± 0.46
*P* value	<0.05	<0.05	<0.05

### Comparison of clinical care satisfaction for patients in both groups

Comparison of clinical care satisfaction for patients in both groups showed that there was significant difference between the two groups (*P*<0.05, Table 4). The patients in the treatment group were more satisfied compared with the control group.

**Table 4:** Assessment of patients’ nursing care satisfaction

**Groups**	**Cases (n)**	**Satisfaction assessment**
Treatment group	55	7.22 ± 1.33
Control group	55	4.15 ± 0.88
*X2* value	-	3.4
*P* value		<0.05

## Discussion

Valvular heart disease or coronary infarction cause significant decreases in patients’ quality of life and their mental stress also gradually increases (4). In recent years, we have been surveying the nursing care experience and we have found that the best efficacy of nursing care can be achieved by not only recording patients’ general physical condition pre-and post-surgery but also by positively grasping patients’ individual psychological state at different stages, thus providing them with personal nursing intervention.

The results of this study showed that the SCL-90 and PSQI scores of cardiothoracic patients after surgery were lower than those before surgery were, thus indicating that a certain degree of psychological nursing intervention generates clinical efficacy for cardiothoracic patients in their rehabilitation. On comparing the obtained experimental data between the two groups, we observed that the graded nursing care applied to the patients in the treatment group generated more significant efficacy than the regular nursing care applied to those in the control group. In clinical practice, the patients in the treatment group clearly showed that their negative feelings had disappeared and sleep quality was significantly improved (5–8).

Based on years of nursing care for cardiothoracic patients, we found that, although sedative drugs could alleviate post-surgical pain, anxiety, and agitation, effective psychological intervention obviously improved patients’ psychological state and comprehension of their disease. It reduced the excessive excitability of patients’ sympathetic nervous system, which was caused by acute trauma, as well as the resulting fluctuation in heart rate, blood pressure, blood glucose, respiratory rate, urine volume, sleep, and feelings. With such nursing care, we could not only reduce patients’ medical costs but also improve their quality of life (8). Unlike regular nursing care, psychological nursing care supplements normal medical practices rather than superseding them. Patients attending Mindfulness-based Stress Reduction (MBSR) often have varying physiological or psychological diseases, such as headache, hypertension, backache, heart diseases, cancer, AIDS, asthma, chronic pains, fibromyalgia, dermatosis, stress-related gastrointestinal diseases, sleep disorders, and anxiety and panic disorders. Before being eligible to apply psychological nursing care, nurses should have worked for years, having accepted standard psychological training and acquiring the qualifications required for psychological counselors. Previous studies have shown that a patient’s healthy psychological state is very important for his/her rehabilitation after cardiothoracic surgery (9–11).

The results of this study demonstrated that individualized graded nursing care is more widely applicable than regular nursing care. Patients vary in their psychological states due to individual differences. If we apply the same nursing care procedure to every patient, personal abnormal feelings may be neglected, particularly those of cardiothoracic patients. Such overlooking could amplify patients’ negative feelings, sometimes even affecting their psychological and physiological states. Applying individualized nursing-care procedures for patients in different psychological states is a specific method that may render better psychological nursing care to patients, accelerate their rehabilitation, and increase the efficiency of nursing care by medical professionals. One limitation of this study was that we could only consider patients’ SCL-90 scores and sleep quality, as the data did not express patients’ degree of anxiety and depression. Thus, we could not determine whether psychological nursing care actually influences rehabilitation. In future studies, we will comprehensively analyze the efficacy of psychological nursing care by observing various indexes, such as physical rehabilitation and heart rate.

As medical technology continues to develop and surgeries become safer, patients are decreasingly worried about and afraid of the surgeries. Nevertheless, surgery presents great risk, especially cardiothoracic surgery, which causes more negative feelings in patients, such as fear, worry, and anxiety. All these factors contribute to the increase in the difficulty of surgery, tension between medical professionals and patients, and obstacles to patients’ rehabilitation. At present, the graded psychological nursing care procedure is greatly significant for patients’ rehabilitation as they may not only ease the strained tension between medical professionals and patients but also mitigate patients’ negative feelings due to their disease. This procedure will thus accelerate their rehabilitation process and indicate a better efficacy than regular psychological nursing care (12–14). The graded psychological nursing care procedure refers to medical professionals applying psychological knowledge and skills to provide individualized care for post-operation cardiothoracic patients with varying emotional conditions to alleviate their negative feelings, make them more confident, and maintain an optimistic attitude through their rehabilitation (15–18).

On comparing the efficacy between regular and graded psychological nursing care, we found that the latter might better improve the negative feelings and promote the rehabilitation of different cardiothoracic patients with varying emotional conditions (19–20).

## Conclusion

Not only did the application of graded psychological nursing care in post-operation cardiothoracic patients improve nursing care efficiency but it also mitigated patients’ negative feelings. Thus, graded psychological nursing care should be promoted in clinical practice.

## Ethical considerations

Ethical issues (Including plagiarism, informed consent, misconduct, data fabrication and/or falsification, double publication and/or submission, redundancy, etc.) have been completely observed by the authors.
